# Significance of spatial organization of chromosomes in the progression of acute myeloid leukemia

**DOI:** 10.1186/s40880-017-0207-6

**Published:** 2017-04-20

**Authors:** Xueli Tian, Yanfang Wang, Dieyan Chen, Xiaoyan Ke, Wanyun Ma

**Affiliations:** 1 State Key Laboratory of Low-Dimensional Quantum Physics, Department of Physics, Tsinghua University, Beijing, 100084 P. R. China; 2 Department of Hematology, Peking University Third Hospital, Beijing, 100191 P. R. China; 3 Collaborative Innovation Center of Quantum Matter, Beijing, 100084 P. R. China

Dear editor

Leukemia ranks as one of the ten most fatal cancers [1]. The mortality and incidence of this disease are associated with multiple factors, including environmental factors, sex, and age. Distinct genetic and chromosomal aberrations differentially affect the phenotype and prognosis of individuals with leukemia [2, 3]. The t(8;21)(q22;q22) translocation, which is observed in patients with acute myeloid leukemia with maturation (AML-M2, according to the French–American–British classification system), is characterized by the fusion of *AML1* (acute myeloid leukemia factor 1, also referred to as *RUNX1* [runt-related transcription factor 1]) on chromosome 21 and *ETO* (eight-twenty-one, also referred to as *RUNX1T1* [runt-related transcription factor 1, translocated to 1]) on chromosome 8. Although the t(8;21)(q22;q22) translocation is associated with a favorable prognosis, relapse remains the primary cause of treatment failure [4]. Real-time fluorescent quantitative polymerase chain reaction (RQ-PCR) is a powerful tool for monitoring the presence of residual disease and planning treatment strategies [5, 6]; however, this technique is not 100% accurate [7]. The three-dimensional organization of chromosomes might be a valuable prognostic marker of the risk of relapse in patients with t(8;21)(q22;q22)-positive AML [8]. To further study the utility of this approach, we evaluated bone marrow (BM) samples from a patient with t(8;21)(q22;q22)-positive AML-M2 before and after hematopoietic stem cell transplantation (HSCT) using three-dimensional fluorescence in situ hybridization (3D-FISH) and confocal laser scanning microscopy to delineate and analyze the spatial organization of the target chromosomes. *AML1*-*ETO* fusion transcripts detected by RQ-PCR were also discussed in this article.

The 35-year-old male patient was diagnosed with t(8;21)(q22;q22)-positive AML-M2 in January 2013 in Peking University Third Hospital. He was treated with induction chemotherapy and achieved the first complete remission (CR1) in March 2013. However, he deteriorated since November 2014 and relapsed in January 2015. The patient received induction chemotherapy again and achieved the second complete remission (CR2) in March 2015. Then he underwent HSCT in June 2015 and relapsed again in November 2015. Using aspiration, BM specimens (4 mL each) were extracted from the patient when he achieved CR2 (CR2 sample), 2 months after HSCT (post-HSCT sample), 3 months after HSCT (follow-up 1 sample), and 2 months after the first follow-up (follow-up 2 sample). The fifth BM sample (relapse) was extracted 2 weeks after the second follow-up, when the patient relapsed.

Mononuclear cells were isolated from the BM specimens using density gradient centrifugation, and total RNA was extracted using TRIzol Reagent (Invitrogen, Carlsbad, CA, USA). Complementary DNA (cDNA) was synthesized using the ProFlex PCR System (Applied Biosystems, Foster City, CA, USA), and RQ-PCR experiments were conducted to detect the *AML1*-*ETO* fusion transcripts using the 7500 Real-time PCR System (Applied Biosystems) and TaqMan technology. Abelson (*ABL*) was used as the reference gene.

**Fig. 1:**
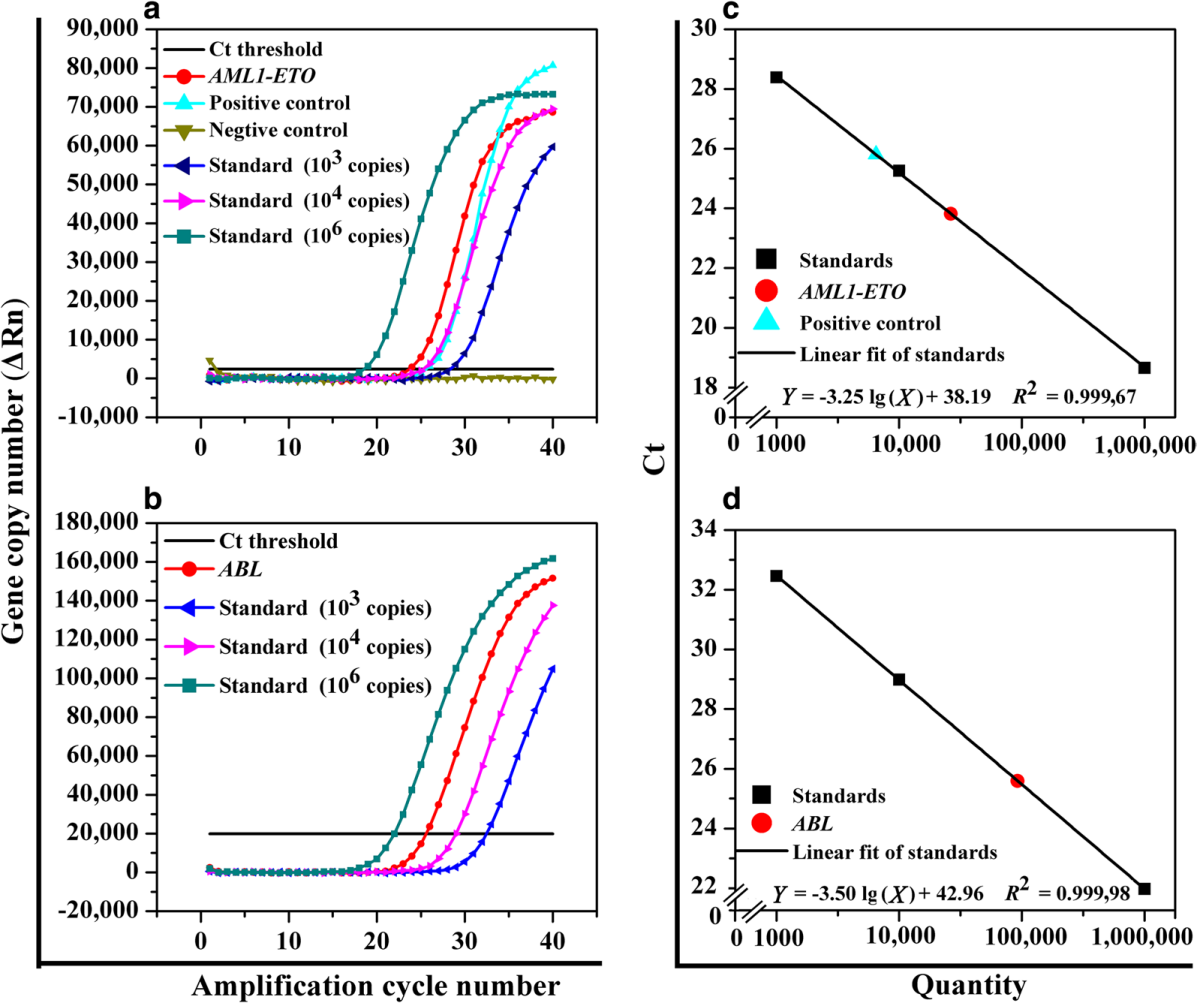
Real-time fluorescent quantitative polymerase chain reaction analysis of the bone marrow sample extracted when the patient with t(8;21)(q22;q22)-positive acute myeloid leukemia with maturation (AML-M2) relapsed after induction chemotherapy and hematopoietic stem cell transplantation. **a** Amplification plots of *AML1*-*ETO* (acute myeloid leukemia factor 1-eight-twenty-one), positive control, negative control, and three standard concentrations (10^3^, 10^4^, and 10^6^ copies) of the *AML1*-*ETO* gene. **b** Amplification plots of *ABL* (Abelson) and three standard concentrations (10^3^, 10^4^, and 10^6^ copies) of the *ABL* gene. **c** Standard curve of the *AML1*-*ETO* gene copy number. *Y* = −3.25lg(*X*) + 38.19, *R* ^2^ = 0.999,67. The negative control gene product was undetectable. **d** Standard curve of the *ABL* gene copy number. *Y* = −3.50lg(*X*) + 42.96, *R* ^2^ = 0.999,98

Representative RQ-PCR curves for the relapse sample were drawn using the OriginPro 8 software (OriginLab Corporation, Northampton, MA, USA) (Fig. 1). The threshold cycle was defined as the cycle number at which the fluorescence surpassed the threshold value. The threshold value was defined as a value greater than the baseline value but sufficiently low enough to lie within the exponential growth region of the amplification curve. The normalized level of *AML1*-*ETO* transcripts was calculated by dividing the total *AML1*-*ETO* copy number by the total *ABL* copy number. *AML1*-*ETO* transcripts were undetectable by RQ-PCR prior to disease relapse. The patient relapsed 5 months after HSCT, during which a rapid increase in *AML1*-*ETO* transcripts was observed (Table 1).

**Table 1:** Results of RQ-PCR for *AML1*-*ETO* fusion transcript detection and 3D-FISH for chromosomal organization detection in a 35-year-old male patient with t(8;21)(q22;q22)-positive AML-M2

Disease course	*AML1*-*ETO* level^a^ detected with RQ-PCR (%)	Percentage of cells detected with 3D-FISH					
		Labeling chromosomes 8 and 21			Labeling chromosomes 8 and 18		
		Normal cells (%)	Proximal cells (%)	Malignant cells (%)	Normal cells (%)	Proximal cells (%)	Malignant cells
CR2	0.1	54.2	41.6	4.2	NA	NA	NA
Post-HSCT	0.0	55.2	29.9	14.9	61.4	30.0	8.6% (8.6% for 3g2r)
Follow-up 1	0.0	42.9	34.9	22.2	46.5	39.4	14.1% (11.3% for 3g2r)
Follow-up 2	0.0	36.1	34.4	29.5	47.9	39.8	12.3% (10.8% for 3g2r)
Relapse	28.6	32.8	29.5	37.7	45.4	47.0	7.6% (6.1% for 3g2r)

RQ-PCR, real-time fluorescent quantitative polymerase chain reaction; *AML1*-*ETO*, acute myeloid leukemia factor 1-eight-twenty-one; 3D-FISH, three-dimensional fluorescence in situ hybridization; AML-M2, acute myeloid leukemia with maturation; CR2, second complete remission; HSCT, hematopoietic stem cell transplantation; 3g2r, three green signals and two red signals, indicating the break of chromosome 8; NA, not applicable (only chromosomes 8 and 21 were labeled and analyzed for the CR2 sample)

^a^Normalized level of *AML1*-*ETO* transcripts was calculated by dividing the total *AML1*-*ETO* copy number by the total *ABL* copy number

After an incubation of 150 min at 37 °C in an environment containing 5% CO_2_, mononuclear cells adhered to two microscope slides, which were used for the spatial chromosomal organization analysis with 3D-FISH. One slide was used to label and analyze chromosomes 8 and 21 (translocation-associated), whereas the other slide was used to label and analyze chromosomes 8 and 18 (translocation-irrelevant) to reconstruct them in situ and analyze their spatial organization. Whole chromosome 8 fluorescein isothiocyanate (FITC)-conjugated probes, whole chromosome 21 tetramethylrhodamine (TRITC)-conjugated probes, and whole chromosome 18 TRITC-conjugated probes (Kreatech Diagnostics, Amsterdam, the Netherlands) were used to label chromosomes 8, 21, and 18, respectively. The nuclei were counterstained with diamidinophenylindole. 3D-FISH was conducted using a ThermoBrite S500 system (StatSpin, Inc., Westwood, MA, USA). The samples were denatured for 5 min at 75 °C, and the probes were hybridized for 48 h at 37 °C. Optical sections were acquired at room temperature using a Nikon A1Rsi confocal microscope (Nikon Corporation, Shinagawa-ku, Tokyo, Japan) equipped with a plan apo 100 ×/1.4 NA oil immersion objective. Images with an 80 nm × 80 nm resolution and 500 nm axial step size were obtained. A minimum of 60 cells from each slide were evaluated.

**Fig. 2:**
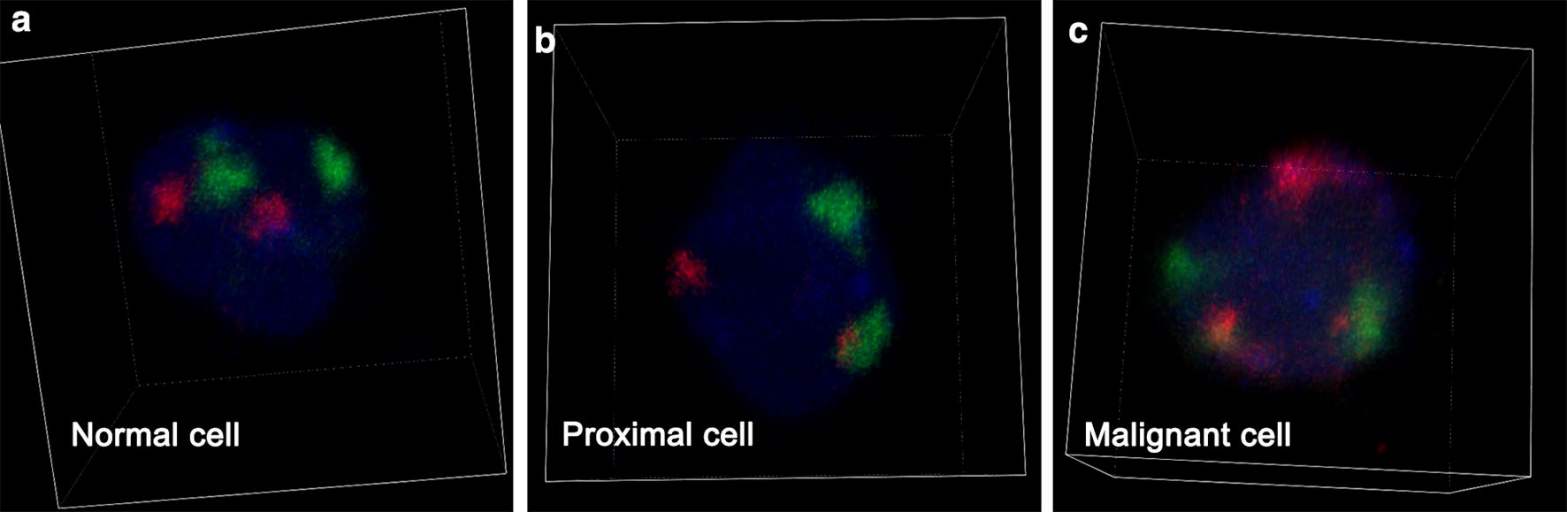
Classification of cells based on the three-dimensional organization of chromosomes 8, 21, and 18. Serial optical sections were acquired using a Nikon A1Rsi confocal system. The images show the three-dimensional reconstruction of nuclei, which are stained blue. *Red signals* indicate either chromosome 21 or 18, and *green signals* indicate chromosome 8. **a** Normal cells. Two green and two red signals are observed in the nuclei. Both copies of chromosome 8 are separate from chromosome 21 and 18. **b** Proximal cells. Two green and two red signals are observed in the nuclei. At least one copy of chromosome 8 is proximal to either chromosome 21 or 18, and the chromosomes are in contact with one another. **c** Malignant cells. Either three green or three red signals are observed in the nuclei. The signals represent chromosome breaks (3g2r or 2g3r) or chromosome translocations (3g3r)

Figure 2 shows the three-dimensional view of the nuclei. The cells were classified as normal, proximal, or malignant cells. Cells with two green and two red signals in the nuclei and in which green and red signals were distantly separate were classified as normal cells (Fig. 2a). Cells with two green and two red signals in the nuclei and in which at least one green and one red signals contacted or fused with each other were classified as proximal cells (Fig. 2b). Cells with three green or three red signals were classified as malignant cells (Fig. 2c). Table 1 shows the percentage of each type of cells in each sample as detected using 3D-FISH. The percentage of malignant cells with aberrations in chromosomes 8 and 21 was significantly higher in the post-HSCT sample than in the CR2 sample (4.2 vs. 14.9%, *P* = 0.033), and increased slowly during follow-up until relapse although without significant difference. Differences in the percentage of malignant cells with aberrations in chromosomes 8 and 18 were not significant among all samples.

**Fig. 3:**
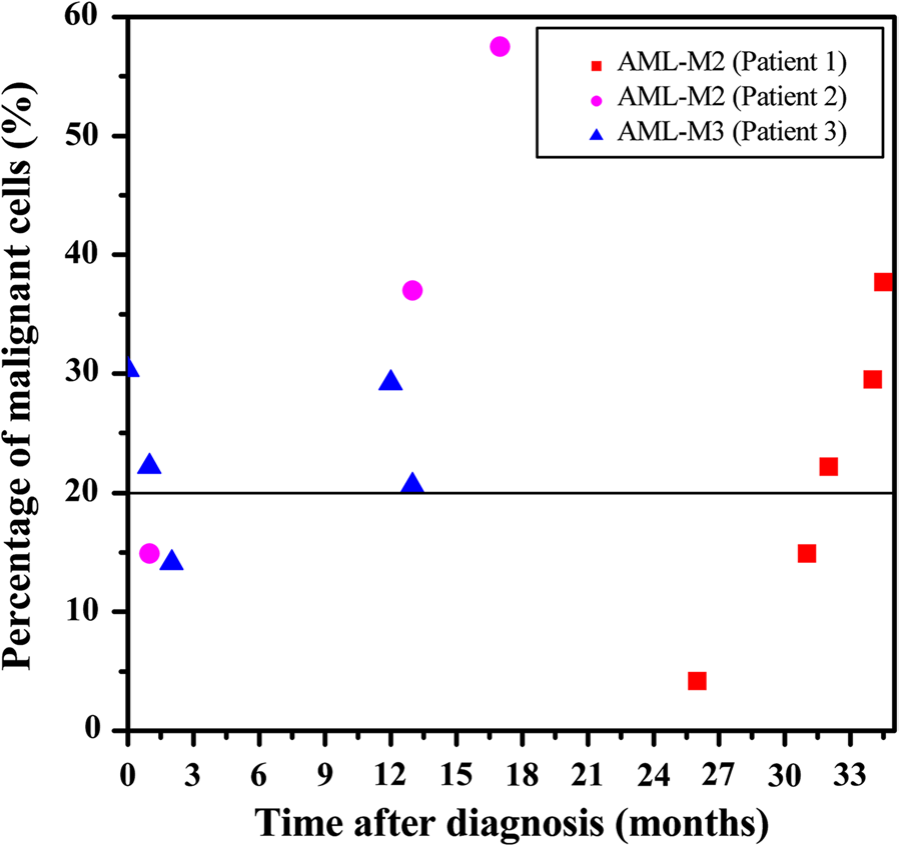
Percentage of malignant cells detected with three-dimensional fluorescence in situ hybridization (3D-FISH) for the three AML patients. The horizontal line represents the probable threshold of 20%, at which point the patient begins to deteriorate. Patient 1 relapsed at 2.5 months and patient 2 relapsed at 4 months after detection of disease deterioration with malignant cells of more than 20%. Patient 3 began to deteriorate in the 12th month after diagnosis, which was indicated by the cerebrospinal fluid containing 10% cancer cells as measured using flow cytometry

In addition to the aforementioned AML-M2 patient, two other AML patients were also treated and followed up at Peking University Third Hospital. Figure 3 shows the percentage of malignant cells detected with 3D-FISH for the three patients. Patient 1 has been discussed above. Patient 2 with t(8;21)(q22;q22)-positive AML-M2 relapsed 17 months after diagnosis. At 4 months before relapse, 3D-FISH analysis showed that the percentage of malignant cells was 37.2%. Patient 3 with t(15;17)(q22;q21)-positive AML-M3 began to deteriorate 12 months after diagnosis, which was indicated by the cerebrospinal fluid containing 10% cancer cells as measured using flow cytometry, and 3D-FISH analysis showed that the percentage of malignant cells was 29.2%.

In our previous study, we evaluated BM samples from t(8;21)(q22;q22)-negative AML-M2 patients and donors with a normal karyotype [8]. The percentage of malignant cells in all participants was <20%. In the first patient described in this study, 3D-FISH demonstrated that 4.2% of cells in the CR2 sample were malignant, indicating that the patient was in complete remission. After HSCT, RQ-PCR showed complete remission with no *AML*-*ETO* fusion transcripts. However, the patient unexpectedly relapsed 5 months after HSCT. 3D-FISH conducted with labeled probes targeting chromosomes 8 and 21 (translocation-associated) demonstrated that the percentage of malignant cells in the BM samples steadily increased after HSCT, indicating that the disease was progressing prior to the time of relapse identification. Among cells labeled with probes targeting chromosomes 8 and 18 (translocation-irrelevant), the percentage of malignant cells detected by 3D-FISH was <20% in all samples, and most malignant cells exhibited chromosome 8 breaks (3g2r in the nuclei) but not chromosome 18 breaks. Furthermore, in cells labeled with probes targeting chromosomes 8 and 21, breaks of both chromosomes were detected. These data indicate that the percentage of malignant cells in the BM samples can contribute to determining the disease prognosis. As shown in Fig. 3, all three patients showed similar phenomenon regarding the significance of malignant cells detected with 3D-FISH as an early warning of potential relapse.

In summary, the spatial organization of chromosomes may play an important role in the progression of AML. As 3D-FISH offers the advantage of labeling and detecting chromosomes in situ and reconstructing them three-dimensionally, it is a useful technique for investigating cancer-associated mechanisms.
